# Items parameters of the space-relations subtest using item response theory

**DOI:** 10.1016/j.dib.2018.06.061

**Published:** 2018-06-26

**Authors:** Farida Agus Setiawati, Rita Eka Izzaty, Veny Hidayat

**Affiliations:** Department of Psychology, Universitas Negeri Yogyakarta, Indonesia

## Abstract

This article describes the item parameters analysis result of the space-relations subtest measurement. This subtest is part of the differential aptitude test (DAT) instrument. The item parameters are characteristic psychometric refer quality of the item. The Item parameters than analyzed in this instrument are item fit model, item difficulty, item discrimination, pseudo-guessing, item information curves, and test information function. The data was collected through documentation technique from the space-relation test conducted at *Biro Psikologi* (Psychology *Bureau*) UNY, amounting to 1046 students from Yogyakarta, Indonesia. Data were analyzed using item response analysis with the assistance of BILOG program.

**Specifications Table**

**Table:** 

**Subject area**	*Psychology*
**More specific subject area**	*Psychometry*
**Type of data**	*Table, curve*
**How data was acquired**	*Documentation from the space-relations test results, consisting of 60 items*
**Data format**	*Analyzed*
**Experimental factors**	*Item value*
**Experimental features**	*Item parameters of instrument consisting of item fit model, item difficulty, item discrimination, pseudo-guessing, item characteristic curve, item information function and test information function.*
**Data source location**	*Special Region of Yogyakarta, Indonesia*
**Data accessibility**	*Data are within this article*

**Value of the data**

• Presents item parameter of the space-relations subtest based on item analysis using item response theory.
• The findings can be used as a reference for researchers or test developers in conducting item selection and creating question compilations, particularly that relates to space relations test.
• This data can be used as a reference in making improvements for items that have inappropriate item parameters.

## 1 **Data**

**Fig. 1:**
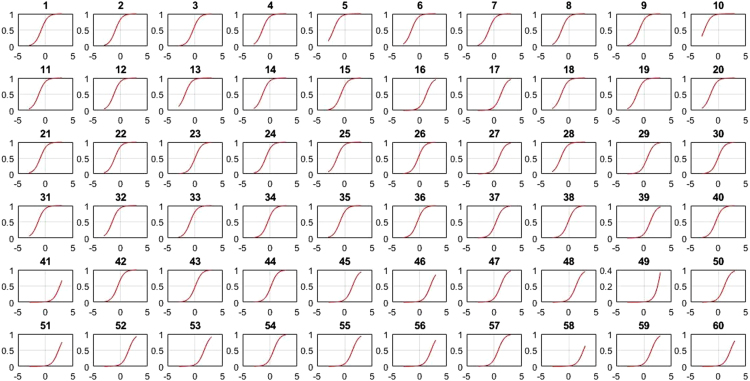
Matrix plot of ICC-1 PL 60 item of space-relation.

**Fig. 2:**
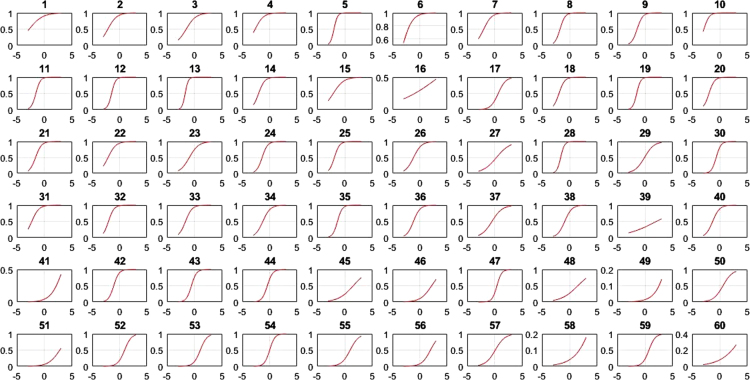
Matrix plot of ICC-2 PL 60 items of space-relation.

**Fig. 3:**
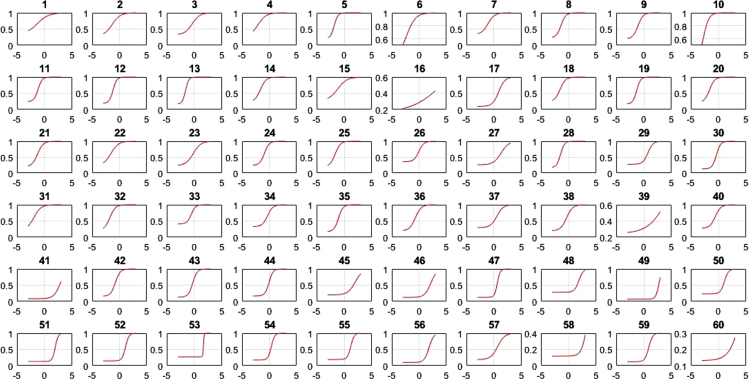
Matrix plot of ICC 3-PL 60 items of space-relation.

This research contains information on the psychometric characteristics of the space-relations sub-test items using items response theory. Item response theory is developed based on two postulates: (1) The examinee performance in a particular test could be predicted using a set of factors called latent traits, where traits are aptitude dimension of a person such as verbal ability, cognitive ability, and so on and (2) The relationship between examinee item performance and the traits that influence it is in accordance with monotonically increasing function called Item Characteristic Curve (1). These values consist of item difficulty (b), item discrimination (a), pseudo-guessing (c), item characteristic curve (ICC), item information function (IIF) and test information function (TIF). Based on the number of item parameter under the study for dichotomous data, there are three logistic models in the modern analysis that could be used; one-parameter (1-PL), two-parameter (2-PL), and three-parameter (3-PL) logistic model [1], [2], [3]. 1-PL model only has one parameter, which is item difficulty level; 2-PL model contains two parameters, i.e. level of item difficulty and discrimination index; while 3-PL model, containing level of difficulty and discrimination index, and pseudo-guessing parameter. Item difficulty shows how difficult an instrument is, judging by its item. Item discrimination index is an item׳s ability to distinguish between a person with high and low-ability to answer questions. Pseudo-guessing refers to the chances that low-ability subjects answer items correctly. Item difficulty, item discrimination and pseudo-guessing were calculated using Bilog program. The model to predict the ability person was calculated by Matlab program of 1,2,3 PL. The presented data derives from item analysis in the form of the fit model and its parameter values. These model were described by item characteristic curve (ICC) formulated 1,2,3. The ICC 1-PL all items of space-relation can be seen in Figs. 1, the ICC all items of 2-PL in Fig. 2, Fig. 3 were ICC of 3 PL.

(1) $$1-\mathrm{PL}\;\mathrm{model}\;\;P_{s}(\theta )\;=\;\frac{e^{D(\theta -b_{i})}}{1\;+\;e^{D(\theta -b_{i})}}$$

(2) $$2-\mathrm{PL}\;\mathrm{model}\;\;P_{s}(\theta )\;=\;\frac{e^{\mathrm{Dai}(\theta -b_{i})}}{1\;+\;e^{\mathrm{Dai}(\theta -b_{i})}}$$

(3) $$3-\mathrm{PL}\;\mathrm{model}\;P_{s}(\theta )\;=\;c_{i}\;+\;(1-c_{i})\frac{e^{\mathrm{Dai}(\theta -b_{i})}}{1\;+\;e^{\mathrm{Dai}(\theta -b_{i})}}$$

**Table:** 

θ	:	Ability person
$P_{s}(\theta )$	:	Probability of success for person s
$a_{i}$	:	Item discrimination
$b_{i}$	:	Item difficulty
$c_{i}$	:	*Pseudo guessing*
*e*	:	Exponential (2718)
*D*	:	Scaling constant (1702)

Item information function (IIF) is a method IRT to explained the item representation, It shows the score accuracy of the items. Birmbaum [1] defined this information in formula 4. Summary of IIF is test information function (TIF), It has the same concept with reliability in classical theory of measurement (4). Based of TIF, could be calculated standard error of measurement based of test. TIF and *Standard Error of measurement* (SEM) were representation the accuracy of test that explained latent variable [1], [2], [6], [7].

(4) $$\;I_{1}(\theta )=\frac{2,89\alpha_{i}^{2}(1-c_{i})}{\left[c_{i}+e^{1,7\alpha (\theta -\beta_{i})}\right]+{\left[1+e^{1,7\alpha (\theta -\beta_{i})}\right]}^{2}}$$

(5) $$\;I(\theta )=\overset{n}{\underset{i=1}{\sum }}I_{i}(\theta )$$

(6) $$\;\mathrm{SEM}(\theta )=\frac{1}{\sqrt{I_{i}(\theta )}}$$

**Table:** 

$I_{i}$(*θ*)	:	Item information function
I(*θ*)	:	Test information function
SEM(*θ*)	:	Standard error of measurement

**Fig. 4:**
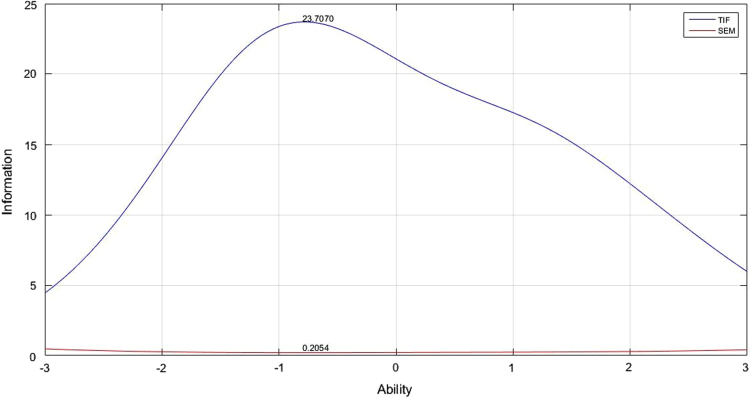
TIF, SEM and their interaction in 1-PL model.

**Fig. 5:**
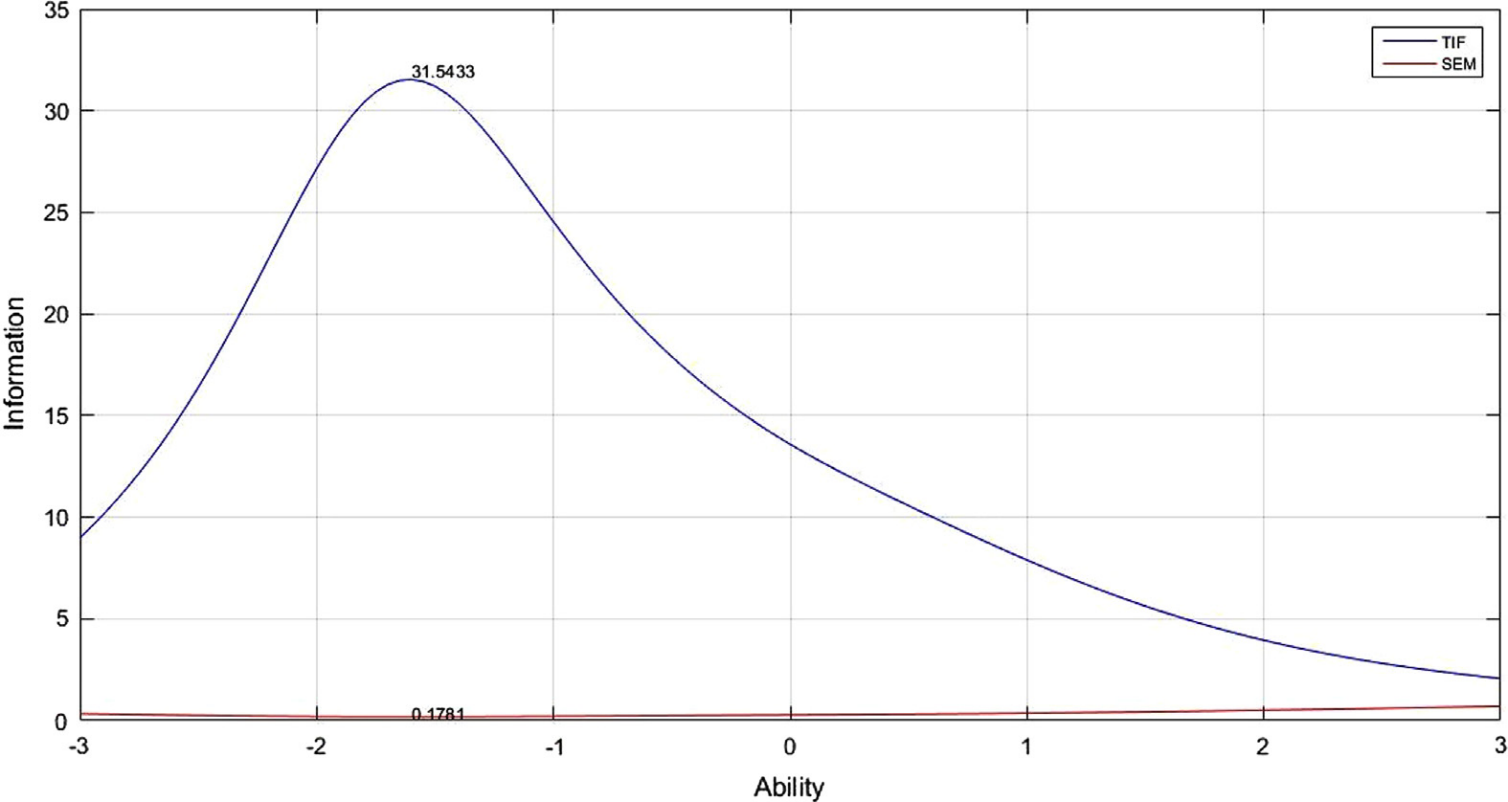
TIF, SEM and their interaction in 2-PL model.

**Fig. 6:**
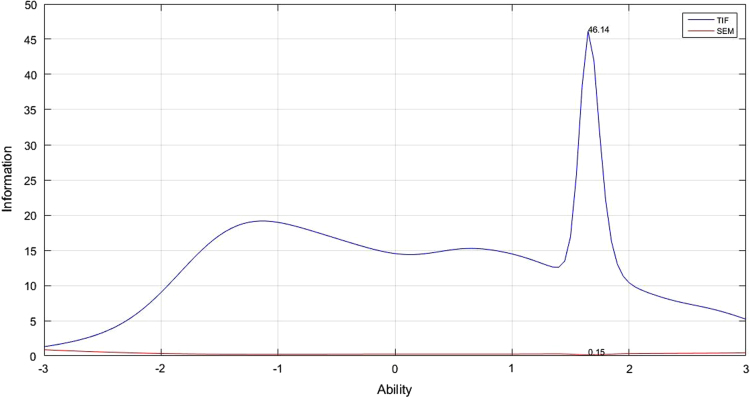
TIF, SEM and their interaction in 3-PL model.

Table 1 depicts the result of the fit model analysis. Table 2 shows data on parameters item, they are item difficulty, item discrimination index, and pseudo-guessing index. Meanwhile, Fig. 1, Fig. 2, Fig. 3 presented the item characteristic curve and Fig. 4, Fig. 5, Fig. 6 showed test information function and standard error of measurement each response theory model.

**Table 1:** Items fit model.

**Items**	**1-PL**	**2-PL**	**3-PL**
1	0.0029	0.6569^*^	0.5294^*^
2	0.3648^*^	0.2521^*^	0.4389^*^
3	0.1831^*^	0.6837^*^	0.4941^*^
4	0.6447^*^	0.1681^*^	0.5795^*^
5	0.0000	0.0981^*^	0.3192^*^
6	0.3782^*^	0.3500^*^	0.9732^*^
7	0.7005^*^	0.3968^*^	0.6683^*^
8	0.0094	0.6277^*^	0.7045^*^
9	0.0433^*^	0.5537^*^	0.6021^*^
10	0.4277^*^	0.9065^*^	0.6990^*^
11	0.0009	0.8785^*^	0.7311^*^
12	0.0000	0.8206^*^	0.9475^*^
13	0.0000	0.1825^*^	0.4329^*^
14	0.5262^*^	0.6649^*^	0.2810^*^
15	0.3797^*^	0.7385^*^	0.6590^*^
16	0.0000	0.0363^*^	0.0860^*^
17	0.6001^*^	0.1377^*^	0.6284^*^
18	0.2235^*^	0.6018^*^	0.2912^*^
19	0.0000	0.1661^*^	0.6191^*^
20	0.0294^*^	0.0542^*^	0.2007^*^
21	0.2362^*^	0.0872^*^	0.2089^*^
22	0.3913^*^	0.5723^*^	0.9742^*^
23	0.4314^*^	0.0710^*^	0.0675^*^
24	0.0094	0.3945^*^	0.9251^*^
25	0.0567^*^	0.3261^*^	0.4144^*^
26	0.4286^*^	0.4423^*^	0.4291^*^
27	0.0440^*^	0.9126^*^	0.9691^*^
28	0.0000	0.9084^*^	0.7518^*^
29	0.0933^*^	0.0552^*^	0.1518^*^
30	0.0001	0.8564^*^	0.9462^*^
31	0.6778^*^	0.3491^*^	0.5254^*^
32	0.2142^*^	0.3184^*^	0.8514^*^
33	0.0011	0.0140^*^	0.0301^*^
34	0.3045^*^	0.3375^*^	0.8175^*^
35	0.0002	0.8522^*^	0.7452^*^
36	0.7344^*^	0.9127^*^	0.9659^*^
37	0.0396^*^	0.3363^*^	0.1837^*^
38	0.0004	0.1175^*^	0.0565^*^
39	0.0000	0.5585^*^	0.7487^*^
40	0.2148^*^	0.7542^*^	0.7650^*^
41	0.4149^*^	0.6215^*^	0.5863^*^
42	0.0018	0.2345^*^	0.1039^*^
43	0.0001	0.8568^*^	0.2961^*^
44	0.0002	0.1759^*^	0.4999^*^
45	0.0349^*^	0.6893^*^	0.9907^*^
46	0.7604^*^	0.6720^*^	0.2277^*^
47	0.0000	0.0293^*^	0.4359^*^
48	0.0000	0.6248^*^	0.8857^*^
49	0.0076	0.3234^*^	0.6020^*^
50	0.0008	0.0364^*^	0.1911^*^
51	0.0008	0.0042^*^	0.2743^*^
52	0.002	0.0246^*^	0.8209^*^
53	0.0000	0.0235^*^	0
54	0.0001	0.2573^*^	0.8223^*^
55	0.0014	0.003	0.3977^*^
56	0.7173^*^	0.8729^*^	0.9058^*^
57	0.8483^*^	0.6158^*^	0.3843^*^
58	0.0000	0.0316^*^	0.5223^*^
59	0.0081	0.0106^*^	0.2373^*^
60	0.0000	0.9888^*^	0.9954^*^

**Table 2:** Items parameters of space-relations test.

**Items**	**Item difficulty (b)**			**Item discrimination (a)**		**Pseudo-guessing index (c)**
	**1-PL**	**2-PL**	**3-PL**	**1-PL**	**2-PL**	
1	−0.783	−2.768^*^	−1.304	0.486	0.577	0.338^*^
2	−0.906	−2.144^*^	−1.338	0.715	0.796	0.285^*^
3	−0.367	−1.522	−0.391	0.654	0.862	0.323^*^
4	−1.384	−2.657^*^	−2.086^*^	0.76	0.788	0.257^*^
5	−2.031^*^	−1.949	−1.817	1.82	1.765	0.209
6	−1.466	−3.125^*^	−2.331^*^	0.656	0.704	0.284^*^
7	−0.981	−2.008^*^	−1.077	0.822	1.009	0.336^*^
8	−1.278	−1.731	−1.366	1.281	1.372	0.23
9	−0.834	−1.427	−1.066	1.166	1.256	0.197
10	−2.522^*^	−2.847^*^	−2.714^*^	1.234	1.17	0.237
11	−1.218	−1.564	−1.185	1.447	1.597	0.242
12	−1.181	−1.455	−1.189	1.602	1.718	0.191
13	−1.82	−1.707	−1.611	2.074	2.011	0.168
14	−1.341	−2.023^*^	−1.629	1.058	1.112	0.228
15	−0.679	−2.075^*^	−1.159	0.618	0.706	0.273^*^
16	1.253	3.455^*^	5.186^*^	0.147	0.195	0.159
17	1.198	0.608	0.85	0.866	1.044	0.095
18	−1.342	−1.915	−1.554	1.148	1.184	0.234
19	−1.261	−1.497	−1.301	1.638	1.668	0.171
20	−1.408	−1.929	−1.598	1.189	1.247	0.215
21	−0.955	−1.622	−1.269	1.077	1.138	0.194
22	−1.036	−2.100^*^	−1.554	0.81	0.862	0.236
23	−0.014	−0.949	−0.194	0.7	0.865	0.233
24	−0.792	−1.384	−0.896	1.171	1.354	0.241
25	−1.402	−1.867	−1.641	1.249	1.232	0.195
26	−0.247	−1.143	−0.051	0.801	1.297	0.355^*^
27	0.829	0.295	1.267	0.48	0.857	0.258^*^
28	−1.452	−1.61	−1.462	1.691	1.652	0.171
29	0.614	−0.089	0.745	0.677	1.283	0.275^*^
30	−0.055	−0.633	−0.368	1.373	1.558	0.131
31	−1.359	−2.297^*^	−1.943	0.901	0.909	0.21
32	−1.482	−2.029^*^	−1.748	1.162	1.164	0.214
33	−0.889	−1.648	−0.566	0.992	1.535	0.409^*^
34	−0.393	−1.255	−0.321	0.859	1.22	0.326^*^
35	−0.862	−1.315	−1.037	1.369	1.455	0.174
36	−0.403	−1.165	−0.69	0.963	1.1	0.199
37	0.329	−0.526	0.514	0.626	1.025	0.295^*^
38	−0.099	−0.88	−0.362	0.901	1.073	0.197
39	1.15	2.060^*^	3.973^*^	0.205	0.351	0.247
40	−0.479	−1.276	−0.563	0.932	1.186	0.274^*^
41	2.587^*^	3.307^*^	2.818^*^	0.545	1.003	0.085
42	−0.479	−1.069	−0.752	1.219	1.347	0.161
43	0.036	−0.578	−0.305	1.292	1.452	0.131
44	0.212	−0.439	−0.048	1.206	1.606	0.17
45	1.409	1.531	2.003^*^	0.443	0.872	0.202
46	1.999	2.156^*^	2.145^*^	0.582	1.042	0.124
47	1.125	0.374	0.595	1.38	2.282^*^	0.126
48	1.299	1.484	1.859	0.392	1.481	0.282
49	3.314^*^	5.272^*^	2.719^*^	0.469	2.018^*^	0.069
50	1.09	0.589	1.092	0.685	1.611	0.235
51	2.356^*^	2.758^*^	1.929	0.58	2.194^*^	0.129
52	1.525	0.924	1.098	0.947	1.888	0.147
53	1.641	1.027	1.636	0.972	9.061^*^	0.27
54	0.693	−0.008	0.367	1.238	1.94	0.172
55	1.421	0.977	1.194	0.753	1.899	0.19
56	2.145^*^	1.95	1.805	0.744	1.362	0.099
57	0.716	0.071	0.672	0.674	0.933	0.187
58	2.670^*^	5.779^*^	3.443^*^	0.321	1.15	0.118
59	1.37	0.709	0.89	1.018	1.673	0.122
60	2.217^*^	5.116^*^	4.851^*^	0.281	0.521	0.131

## 2 **Experimental design, materials and methods**

This study aims to analyze the data documentation of psychological test results, namely space-relations. The findings could be used to evaluate existing measuring tools and developing it into a new format. Item parameter analysis of the instrument was done using a modern approach, namely item response theory (IRT). IRT is the analysis of characteristic instrument that focused on information item [1], [2], [3], [4].

The research instrument is a space-relation test, part of a differential aptitude test (DAT). This instrument was adapted in Indonesia. The original instrument was composed by Bennet, Harold G. Seashore, & Wesman in 1947 [8]. This instrument is in the form of multiple choices question and consists of 60 items that must be done within 30 min. The raw data are responses derived from 1046 subjects in Yogyakarta Special Region (DIY). All data were analyzed using item response theory with the help of BILOG-MG and Matlab program. The BILOG-MG analysis was conducted three times because, based on the number of item parameters studied for dichotomous data types, there are three logistic models in the analysis of item response theory: one parameter logistic model (1-PL), two parameters (2-PL) and three parameters (3-PL). The 1-PL model only contains one item parameter, namely item difficulty; 2-PL model contains two parameters, specifically item difficulty and item discrimination index; while the 3-PL model also includes the pseudo-guessing parameter on top of the previous two [9], [10], [11]. An item-match analysis is performed to ensure which logistic model fits best with the space-relation subtest data (see Table 1). In Table 1, items that match their logistic model (fit model) are marked with *. An item is said to match its logistic model if it has a probability value of ≥ 0.01. The significance level (α) = 0.01 is the minimum fault limit value with the degree of freedom (df) defined in this study [12].

An item is considered appropriate if it meets the item parameter criterion below:

1. A good level of item difficulty is at -2 to +2 [3], [5]. The results of the difficulty analysis are presented in Table 2. Easy items are marked with * and difficult items marked with **. Meanwhile, unmarked items have a moderate degree of difficulty.
2. A good item discrimination index criterion would be above 0 below 2 [3]. Table 2 presents result of the discrimination index analysis. Almost all items have a good discrimination index when analyzed with 2 PL models. There are 4 items need to be evaluated namely item numbers 47, 49, 51, 59.
3. The pseudo-guessing index in the multiple-choice test is around one to the number of available answer choices [13], [14]. Since the number of alternative answers to the space-relation subtest is 4, the maximum value of c is either 1/4 or 0.25. Of the 60 items in the space-relation test, there are 14 items with a high pseudo-guess score, namely item numbers 1, 2, 3, 4, 6, 7, 15, 26, 27, 29, 33, 34, 37 and 40.

The analysis of test information function and the standard error of measurement (SEM) did by Matlab program. The information function of space-relations measurement test analyzed with 1, 2, and 3 logistic parameters. Test information function is described as having a low curve that initially increase, reaching the highest score in the middle before it goes down again away from the midpoint. The width of the curve shows the breadth of effective ability applied from the measurement results. TIF will be effective if the curve line extends above SEM line without having a cutoff point. Fig. 4, Fig. 5, Fig. 6 illustrate TIF, SEM and interaction between them. The three images show the TIF curve to be above SEM without any cutting point, meaning that all information obtained from the measurement results will be accurate on all abilities. Maximum measurement results differ between parameter models. In the 1 PL analysis, the maximum information function is 23,7070 which is in the 0.2054 abilities; the maximum 2 PL information function is 31.5433 with abilities at 0.1781, while the 3-PL information function maximal score is 46.14 with abilities at 0.15.
