# Supplementary material for: Items parameters of the space-relations subtest using item response theory
Source: Data Brief. 2018 Jun 26;19:1785–93. doi: 10.1016/j.dib.2018.06.061 (PMC6141381; doi:10.1016/j.dib.2018.06.061)
Supplement: Supplementary file 1 — Supplementary material [file mmc1.docx]

**COMPLIANCE WITH ETHICAL STANDARDS**

**Items Parameters of the Space-Relations Subtest using**

**Item Response Theory**

Farida Agus Setiawati^1^, Rita Eka Izzaty^2^, Veny Hidayat^3^

^1^ Department of Psychology, Universitas Negeri Yogyakarta

^2^ Department of Psychology, Universitas Negeri Yogyakarta

^3^ Department of Psychology, Universitas Negeri Yogyakarta

**Funding:** The research is funded by Ministry of Research, Technology and Higher Education, Indonesia.

**Conflict of Interest:** The authors declare that they have no conflict of interest.

**Informed Consent:** The data was collected through documentation technique from the space-relation test conducted at *Biro Psikologi* (Psychology *Bureau*) Universitas Negeri Yogyakarta (UNY). Informed consent was obtained from head of Psychology Laboratory UNY.
